# Integrated Morphological and Transcriptomic Analyses of Gene Regulatory Mechanisms in Different Intra-Puparial Developmental Stages of *Phormia regina*

**DOI:** 10.3390/insects17060642

**Published:** 2026-06-17

**Authors:** Jiani Yang, Ruonan Zhang, Rui Zhu, Lan Gao, Chenbin Wang, Zhiya Gu, Yu Wang

**Affiliations:** 1 Department of Forensic Medicine, Soochow University, Ganjiang East Road, Suzhou 215000, Chinanorah0601@163.com (R.Z.); 2 Criminal Police Branch, Shizuishan Public Security Bureau, Shengli East Road, Shizuishan 753299, China; zr.55@163.com (R.Z.); gaolan1028@163.com (L.G.); 3 Key Laboratory of Biodiversity and Biosafety, Nanjing Institute of Environmental Sciences, Ministry of Ecology and Environment, Jiangwangmiao Street, Nanjing 210042, China; wangchenbin@nies.org

**Keywords:** postmortem interval, *Phormia regina*, transcriptome, morphology

## Abstract

*Phormia regina* (Meigen, 1826) (Diptera: Calliphoridae), a necrophagous blow fly of significant forensic value, plays a crucial role in postmortem interval (PMI) estimation. The intra-puparial period of this species constitutes approximately half of its entire immature developmental duration. Therefore, precise aging of intra-puparial specimens is essential to improve the accuracy of PMI estimation. This study was performed at a constant 25 °C, examining intra-puparial morphology, histological sections, and body weight across different developmental stages. Additionally, full-length transcriptome profiling was conducted via a hybrid sequencing strategy combining PacBio single-molecule real-time (SMRT) sequencing and Illumina short-read next-generation sequencing (NGS). Morphological studies revealed the intra-puparial morphological characteristics, the reconstruction process of tissues and organs, and the continuous body weight loss during the intra-puparial period. Transcriptomic research identified a total of 425,349 full-length non-chimeric (FLNC) sequences. After redundancy removal, 84,852 transcript sequences were obtained, of which 46,325 transcripts were annotated. Using day-0 puparia (D0) as the control, differentially expressed gene (DEG) analysis was performed on samples from subsequent developmental stages (D1–D5), identifying 4242, 7964, 9509, 10,526, and 10,011 DEGs, respectively. Results from GO enrichment and KEGG pathway analyses provided reasonable explanations for the behavioral traits of different developmental stages. Eight candidate genes for intra-puparial development were screened, most of which were highly expressed at different time points during the intra-puparial period. Quantitative real-time PCR (qRT-PCR) results showed consistent gene expression trends with the RNA-seq data, confirming the reliability of the RNA-seq results. This study provides key morphological and molecular markers for *P. regina* in forensic PMI estimation.

## Simple Summary

*Phormia regina* is a common insect that helps postmortem interval (PMI) estimation in forensic cases. The intra-puparial period takes nearly half of its development time, so knowing the exact age of puparia is critical for accurate PMI estimation. In this study, we tracked the morphological, histological, and body weight changes in *P. regina* during intra-puparial development and characterized developmental gene expression via full-length transcriptome sequencing at 25 °C. Marked morphological and pigment shifts inside puparia and progressive body weight loss were detected, together with abundant stage-specific expressed genes. Core genes with consistent temporal expression were screened and verified by quantitative real-time PCR (qRT-PCR). The obtained morphological and molecular markers improve the reliability of *P. regina*-based PMI prediction for forensic casework.

## 1. Introduction

Accurate estimation of the postmortem interval (PMI) is critical for criminal investigations [1,2,3]. Within 1–3 days after death, forensic pathological features such as algor mortis, rigor mortis, and livor mortis can be used for PMI estimation. However, as decomposition progresses, these features gradually fade and disappear, reducing the accuracy of traditional pathological methods [4]. Forensic entomology is a branch of forensic science that utilizes the study of insects to assist in criminal investigations [5]. In contrast, entomological evidence can appear within minutes after death and persist on the corpse over months, providing a reliable basis for PMI estimation over extended periods [6]. Therefore, necrophagous insects are often primary evidence for PMI estimation in decomposed remains [7].

Diptera are among the first colonizers of corpses, particularly species from the Calliphoridae family, which can arrive and colonize a body within minutes [8]. Their offspring develop in association with the corpse, providing valuable information for PMI estimation [9]. *Phormia regina* (Meigen, 1826), a species within Calliphoridae, is distributed across the Holarctic region. It is frequently found in colder areas such as North Asia [10], North America [11], and Northern Europe [12], and is a dominant colonizer of human and animal remains [6]. *Phormia regina* can complete its development at temperatures ranging from 10 °C to 41 °C [13,14], and seasonal variations have little impact on its frequency of discovery at crime scenes [15]. This characteristic makes *P. regina* an ideal indicator species for estimating PMI.

Within the life cycle of *P. regina*, the intra-puparial period accounts for about half of the total development time, representing a critical developmental phase [16]. However, accurately determining the developmental progress during the intra-puparial period presents significant challenges. Obvious puparial color changes occur only within several hours after pupariation, and beyond this point, subsequent developmental stages cannot be precisely discriminated based merely on external puparial morphology [17,18]. Intensive metamorphic remodeling proceeds inside the puparium across intra-puparial development, during which larval tissues degrade and reorganize into adult structures alongside sustained energy consumption. Such progressive internal changes trigger regular shifts in morphology, histology and body mass, all of which serve as promising phenotypic markers supporting intra-puparial age estimation of *P. regina*.

Morphological observation via puparium dissection to document sequential external developmental changes is the most widely used classic method for intra-puparial age estimation. Extensive studies have been conducted on forensically relevant sarcosaprophagous flies, including *Chrysomya rufifacies* [19], *Lucilia sericata* [20], *Sarconesia chlorogaster* [21], *Chrysomya albiceps* [22], *Musca domestica* [23] and *Hydrotaea aenescens* [24]. However, superficial observation after puparium removal cannot clarify intra-puparial tissue remodeling, and histological sectioning has therefore been employed to characterize internal developmental features [25]. Although morphological approaches are intuitive, easy to operate, and require no expensive equipment, their outcomes are prone to subjective bias from researchers, particularly during the grading of puparial pigmentation [26]. Transcriptomic analysis effectively offsets these drawbacks because gene expression data avoid human subjective interference and feature high objectivity [27]. Previous researchers applied MACE (Massive Analysis of cDNA Ends) to profile transcriptomes across 15 successive intra-puparial developmental stages of *C*. *vicina* and established a high-resolution developmental gene expression catalog [28], and the screened stage-specific differentially expressed genes represent promising molecular biomarkers for PMI estimation. However, comparable full-length transcriptome resources across continuous intra-puparial developmental stages remain unavailable for *P. regina*. Combined morphological phenotyping and transcriptomic analysis enables preliminary developmental staging via macroscopic traits and subsequent high-precision age calibration with molecular markers, which greatly improves the accuracy of intra-puparial age and PMI estimation and merits further systematic research.

As temperature dominates insect development, 25 °C is commonly used as a reference temperature in laboratory developmental studies of forensic insects [28]. Being a benchmark condition routinely applied in forensic insect developmental research, it ensures robust experimental representativeness and comparability for referencing previous studies [14]. Hence, a constant 25 °C was selected herein to integrate morphological phenotyping and full-length transcriptome profiling for exploring intra-puparial developmental features and molecular regulation of *P. regina*. It aims to elucidate morphological indicators and gene expression patterns across different intra-puparial developmental stages, identify stage-specific markers, and provide a molecular basis for understanding the growth and development of this species.

## 2. Materials and Methods

### 2.1. Sample Collection

*Phormia regina* specimens were originally sampled in July 2021 from Shizuishan, Ningxia, China (38°98′ N, 106°52′ E). Species identification was accomplished via morphological examination following the adult taxonomic key published by Fan [29], combined with molecular verification. The experimental insects originated from a laboratory-bred colony maintained over successive generations. Detailed protocols for colony establishment were documented by Zhang et al. [30].

Fresh pork (20 g) was placed on a 10 × 10 cm^2^ Petri dish as an oviposition substrate and introduced into the adult rearing cage to induce egg-laying. After one hour, the dish was removed, and approximately 200 eggs were observed on the pork. The dish was then transferred to a 30 × 20 × 10 cm^3^ rearing box. The box was placed in an LHP-300H micro-environmental incubator (Suzhou Yingmin Scientific Instrument Co., Ltd., Suzhou, China) set at 25 °C, 75% relative humidity, and a L12:D12 photoperiod. Larval growth was monitored daily, and an adequate amount of pork was added. Upon observing wandering behavior in the larvae, food provision was stopped, and vermiculite was added as a pupariation substrate. Observations were made every 8 h until pupariation occurred. Sampling began after pupariation for the following studies. Owing to negligible body size divergence between male and female intra-puparial individuals of this species, sexes were not separated during sampling. All specimens used across the entire experiment were progeny derived from a single cohort of parent adults.

### 2.2. Weight Measurement

After pupariation, an independent cohort of 77 intact live pupae was collected for bulk weight measurement. Prior to weighing, the analytical balance was fully calibrated; all specimens were transferred onto clean weighing paper, and the entire batch was weighed in three independent technical replicates. The arithmetic average of three repeated measurements was adopted to reduce instrumental and operational weighing error. Bulk measurement rather than single-individual weighing was chosen because individual pupae exhibit extremely low body mass that easily causes large weighing bias, and single-specimen data lacks population representativeness; pooled bulk weight better reflects the actual developmental characteristics of the tested cohort. Measurements started at 0 h and were repeated at 24-h intervals until the first adult emergence.

### 2.3. Intra-Puparial Morphological Observation

Larvae were reared using the methods above until pupariation. Starting from pupariation, 8 pupae were sampled every 24 h until adult emergence. The collected pupae were first placed in 90 °C hot water for 1 min, then preserved in an 80% ethanol solution. All morphological dissection, observation and image capture were performed on an optical microscope (Carl Zeiss, Oberkochen, Germany) at a fixed magnification of 8× under standardized illumination and imaging parameters across all samples. With insect pins and ophthalmic forceps, the cephalic portion of each puparium was peeled open first, and the residual puparial shell was then cut circumferentially for complete removal. Intra-puparial morphological changes at each time point were observed, recorded, and photographed with a camera (Nikon D700, Nikon Corporation, Tokyo, Japan).

For each developmental time point, intact specimens with consistent puparial pigmentation and body dimensions were prioritized as representative individuals, whereas malformed samples featuring incomplete puparium formation or aberrant pigmentation were discarded to avoid phenotypic interference. To reduce subjective observational bias, morphological staging was independently assessed in a blinded manner by two experienced researchers who were blinded to the corresponding developmental age. Any inconsistent scoring results between the two investigators were further discussed and adjudicated by a senior expert to reach a unified conclusion. In addition, all intra-puparial anatomical observations were finished within one week post-sample preservation to prevent tissue degradation-induced morphological distortion.

### 2.4. Intra-Puparial Tissue Sectioning and Staining

According to the developmental data of *P. regina* by Zhang et al. [30], its complete intra-puparial development from pupariation to adult emergence takes approximately 120–130 h, which is the total developmental duration corresponding to our D0–D5 sampling window. We established six sampling time points (D0 to D5) to cover the full developmental process, with three biological replicates per time point (18 specimens in total). All specimens were initially immersed in hot water at 90 °C for 1 min. For D0 samples, four small holes were pricked on the puparium surface using an insect pin. For D1–D5 samples, the entire puparium was removed. All samples were fixed in 75% ethanol for 24 h, then transferred to formalin fixative for another 24 h. The fixed samples were transferred to 60 °C paraffin for immersion, then moved into molds to allow the paraffin to cool and solidify. An embedding machine was used to embed the tissue. A microtome was used to slice the embedded tissue into sections 5–10 micrometers thick. The sections were placed in xylene to remove the paraffin; this process was repeated twice, each lasting 10–15 min. The sections were gradually transferred through a graded alcohol series (100%, 95%, 70%) and finally into distilled water for rehydration. Sections were immersed in hematoxylin stain for 10 min, then in 0.1% acid alcohol to remove excess hematoxylin. Subsequently, sections were immersed in eosin stain for 5 min. After eosin staining, sections were dehydrated again through a graded alcohol series (70%, 95%, 100%) to remove water. Xylene was used for cleaning to remove alcohol. Finally, sections were mounted with mounting medium and covered with a coverslip. They were gently flattened and allowed to solidify for subsequent slide scanning and microscopic observation.

### 2.5. RNA Extraction

Larvae were reared using the methods above until pupariation. Starting from pupariation, samples were collected every 24 h until emergence. Seven pupae were sampled at each time point and immediately frozen at −80 °C. For each developmental stage (D0–D5), three biological replicates were prepared, each replicate consisting of seven pupae pooled together for RNA extraction. Day 1 samples were labeled PR1 (PR1-1, PR1-2, PR1-3), Day 2 as PR2 (PR2-1, PR2-2, PR2-3), and so on. Total RNA from the 18 samples was extracted using Trizol RNA extraction reagent (Invitrogen, Carlsbad, CA, USA). RNA concentration and purity were detected using a Nano Drop 2000c spectrophotometer (Thermo Fisher Scientific, Waltham, MA, USA). A 2% agarose gel was prepared by dissolving 1 g agarose in 50 mL of 0.5× TBE, heating in a microwave until completely melted, cooling to about 60 °C, adding 5 μL EB, mixing, and pouring into a gel tray with a comb. The prepared gel was immersed in an electrophoresis tank filled with 0.5× TBE buffer. A 5 μL aliquot of template mixed with 2 μL 6× loading buffer was loaded per well. Electrophoresis was performed at 150 V for 25 min. After electrophoresis, results were observed on a UV transilluminator and saved. RNA integrity was assessed using a 2100 Bioanalyzer (Agilent Technologies, Santa Clara, CA, USA). An RNA Integrity Number (RIN) greater than 7.0 was considered suitable for library construction.

### 2.6. Transcriptome Sequencing and Differential Gene Expression Analysis

For Illumina HiSeq4000 library construction, six developmental time points (D0–D5) were included with three independent biological replicates per time point, and each replicate was prepared from total RNA isolated from seven pupae. No RNA pooling across time points or replicates was applied for Illumina samples; independent cDNA libraries were constructed for every replicate to preserve biological replicates for subsequent differential expression analysis. For PacBio RS II library preparation, equal amounts of qualified total RNA from all D0–D5 biological replicates were pooled into a single full-length cDNA library. Library construction and sequencing for both platforms were completed at Biomarker Technologies Co., Ltd. (Beijing, China), with the PacBio library sequenced on PacBio RS II and all individual Illumina libraries sequenced on Illumina HiSeq4000 (Biomarker Technologies Co., Ltd., Beijing, China).

The Illumina cDNA libraries were constructed with an insert size of 200–300 bp and sequenced using a paired-end 150 bp (PE150) strategy, with an average sequencing depth of 6 Gb clean data per biological sample. For PacBio full-length transcriptome sequencing, the library insert size was set to 1–6 kb, and single-molecule long-read sequencing was performed to obtain comprehensive full-length transcript information across all developmental stages.

Raw Illumina reads were processed via in-house Perl scripts to remove adapters, sequences containing poly-N stretches, and low-quality reads. Clean data with Q30 > 85% across all samples were retained for subsequent analyses to guarantee data reliability. The non-redundant full-length transcriptome derived from pooled PacBio data was used as the reference genome; no de novo assembly was conducted with Illumina short reads. Clean reads from the 18 independent Illumina libraries (6 stages × 3 biological replicates) were mapped to the PacBio reference using STAR (v2.5.0b) under default parameters. Transcript abundance was quantified with Kallisto (v0.46.1) and normalized to FPKM (fragments per kilobase of transcript per million mapped reads).

To assess sample grouping and similarity, principal component analysis (PCA) was implemented using the factoextra package in R (v3.5.2), and inter-sample correlation analysis was performed with the corrplot package. Differentially expressed genes (DEGs) were screened via DESeq2 (v1.44.0), with thresholds set as FDR ≤ 0.05 and |log_2_(fold change)| ≥ 1.

### 2.7. Clustering and Time Series Analysis

We adopted day-0 puparia (D0) as the control to mine differentially expressed genes for each subsequent developmental day (D1–D5). Significantly differentially expressed genes from all time points were integrated for time series analysis. Genes with excessively low expression or minimal changes across time points were removed. Genes with low standard deviation (low variation between samples) were filtered out. The clustering parameter was set to nine. The optimal fuzzifier value was evaluated using a function, and cluster analysis was performed using the Mfuzz clustering method, which employs the complete linkage algorithm. Gene membership to clusters was determined based on the membership value between genes and clusters. The core function was used to calculate the importance of genes within each trend cluster, helping to select the most representative genes based on membership, yielding visual results.

### 2.8. Gene Annotation and Analysis

Gene function was annotated by referencing the NR, Pfam, KOG/COG/eggNOG, Swiss-Prot, KEGG, and GO databases. The clusterProfiler R package (v4.12.6) was used to perform GO and KEGG enrichment analysis on DEGs to understand involved signaling pathways or biological processes. GO terms and KEGG pathways with a *p*-value ≤ 0.05 were considered significantly enriched.

### 2.9. qRT-PCR Validation

To validate the RNA-Seq results, we adopted an independently reared cohort of *P. regina* under the rearing conditions described above. Samples were collected every 24 h from D0 to D5 according to the aforementioned developmental criteria; total RNA was isolated for qRT-PCR to guarantee consistent developmental progression relative to the original RNA-seq specimens. Primers for quantitative real-time PCR (qRT-PCR) were designed using Primer Premier 5.0 (Premier Biosoft Intl., Palo Alto, CA, USA). The internal reference gene was 60S ribosomal protein L23 (RPL23). Eight target genes were selected: myosin regulatory light chain 2 (MYL2), Chitinase 10 (Cht10), neural-cadherin (CDH2), opsin Rh1 (NINAE), zinc-type alcohol dehydrogenase-like protein C1773 (SPBC1773.06c), TBC1 domain family member 5 (TBC1D5), Fibroblast growth factor receptor-like protein 2 (FGFR2), and disheveled-associated activator of morphogenesis 1 (DAAM1) (Table 1).

Then, 1 μg of RNA was reverse-transcribed into cDNA using HiScript III RT SuperMix (Vazyme Biotech Co., Ltd., Nanjing, China). Finally, a 20 μL reaction mixture, containing 10 μL SYBR Green qPCR mixture (Vazyme Biotech Co., Ltd., Nanjing, China), 1 μL cDNA, 0.4 μL each of forward and reverse primers, and 8.2 μL ddH_2_O, was prepared. QRT-PCR was performed on a LightCycler 96 system (Roche Inc., Branchburg, NJ, USA). The reaction cycle was: pre-denaturation at 95 °C for 30 s, followed by 40 cycles of 5 s at 95 °C and 30 s at 60 °C, with a final step at 95 °C for 10 s. The relative expression of genes was calculated using the 2^−ΔΔCt^ method.

## 3. Results

### 3.1. Body Weight

**Figure 1:**
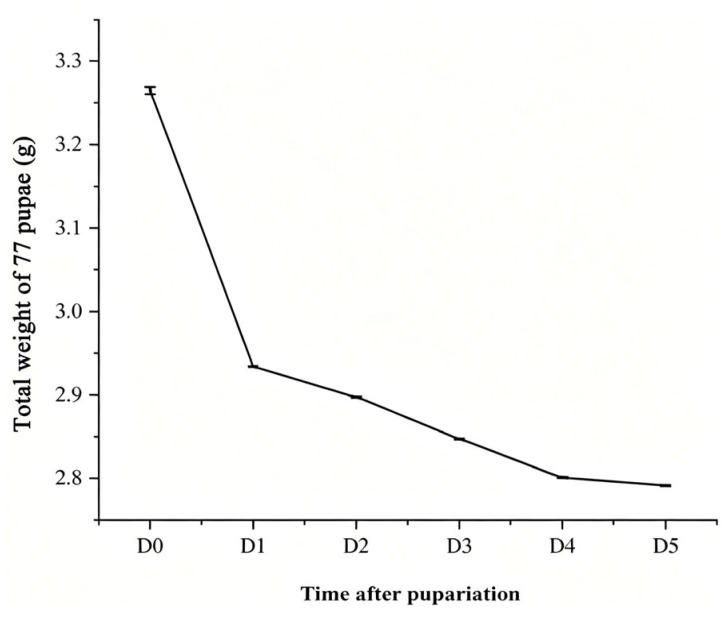
Dynamic total weight changes during the intra-puparial developmental stage of *Phormia regina*. At each sampling time point, an independent batch of 77 healthy pupae was collected for bulk weighing. The error bars represent the standard deviation (SD) of three repeated bulk measurements.

Total weight measurements of 77 pupae exhibited a continuous declining trend across the entire intra-puparial period. As shown in Figure 1, the most prominent weight reduction took place in the early puparial stage, falling from 3.2650 g on D0 to 2.9337 g on D1. This sharp weight loss corresponded to intense energy expenditure driven by extensive initial tissue remodeling during larval-pupal metamorphosis. Thereafter, weight loss slowed markedly from D1 to D5, with total weights recorded as 2.8573 g (D2), 2.8469 g (D3), 2.8012 g (D4) and 2.7908 g (D5), indicative of gradual metabolic consumption and subtle refinement of internal tissues.

### 3.2. Intra-Puparial Morphological Characteristics

*Phormia regina* pupae were reared at a constant 25 °C for 5 days, and morphological characteristics from D0 to D5 were observed.

**Figure 2:**
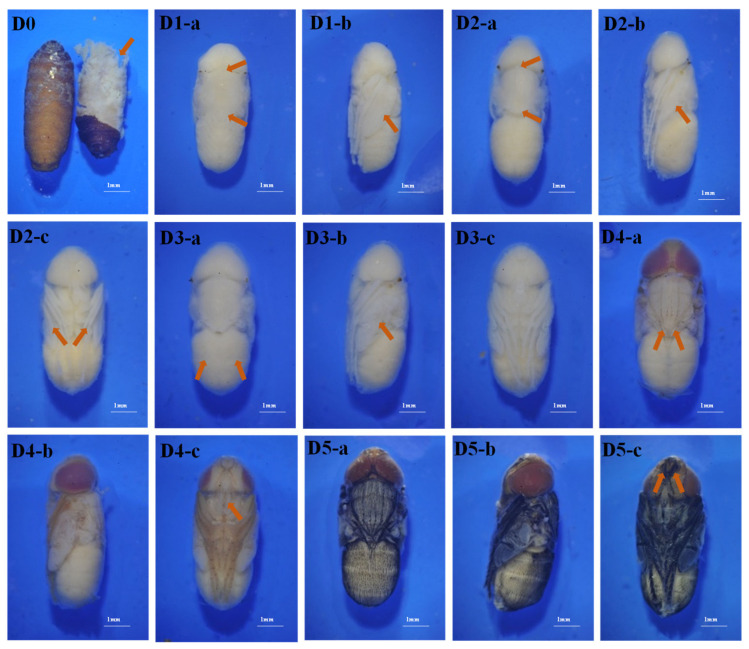
Intra-puparial morphological changes in *Phormia regina* across intra-puparial developmental stages D0–D5 at a constant temperature of 25 °C. (**a**) Dorsal view; (**b**) Lateral view; (**c**) Ventral view. Scale bars = 1 mm. Arrows in (**D0**) indicate tissue damage inside the puparium induced during puparial shell dissection. Arrow in (**D1-a**) marks indistinct demarcation between the head and thorax; (**D1-b**) shows thick wings. In (**D2-a**), clear boundaries among the head, thorax and abdomen are observed; (**D2-b**) presents thin wings and (**D2-c**) slender legs. (**D3-a**) displays segment formation on the abdomen; (**D3-b**) features fully folded wings. (**D4-a**) shows light brown thoracic bristles, and (**D4-c**) has a light-brown labrum; (**D5-c**) bears grayish-black antennae.

D0 intra-puparial morphology: The puparium had just formed, appearing light yellow to light brown. The puparium was tightly adhered to the internal structures, causing damage to internal tissues during separation (Figure 2(D0)).

D1 intra-puparial morphology: Head, thorax and abdomen had differentiated with no distinct demarcation between segments. Legs and wings were visible, and the intra-puparial body was covered with a transparent film. The outlines of the three pairs of legs and one pair of wings were clear. The legs were thick and long, the wings were thick, and they began to extend and differentiate into segments (Figure 2(D1-a–D1-b)).

D2 intra-puparial morphology: The boundaries between the head, thorax, and abdomen became more distinct. Antennae and mouthparts were visible. The legs and wings became thinner (Figure 2(D2-a–D2-c)).

D3 intra-puparial morphology: The wings were completely folded. White bristles on the thorax were visible, extending beyond the thoracic end. Abdominal segmentation was visible (Figure 2(D3-a–D3-c)).

D4 intra-puparial morphology: The compound eyes were purplish-red. Head setae were brown, the labrum was light brown, thoracic bristles were brown, wings were light brown, and the edges of the legs were brown or dark brown (Figure 2(D4-a–D4-c)).

D5 intra-puparial morphology: Antennae were grayish black, compound eyes were brownish-red, the labrum was dark brown, wings were dark gray, and the legs were entirely black (Figure 2(D5-a–D5-c)).

### 3.3. Tissue Sections

**Figure 3:**
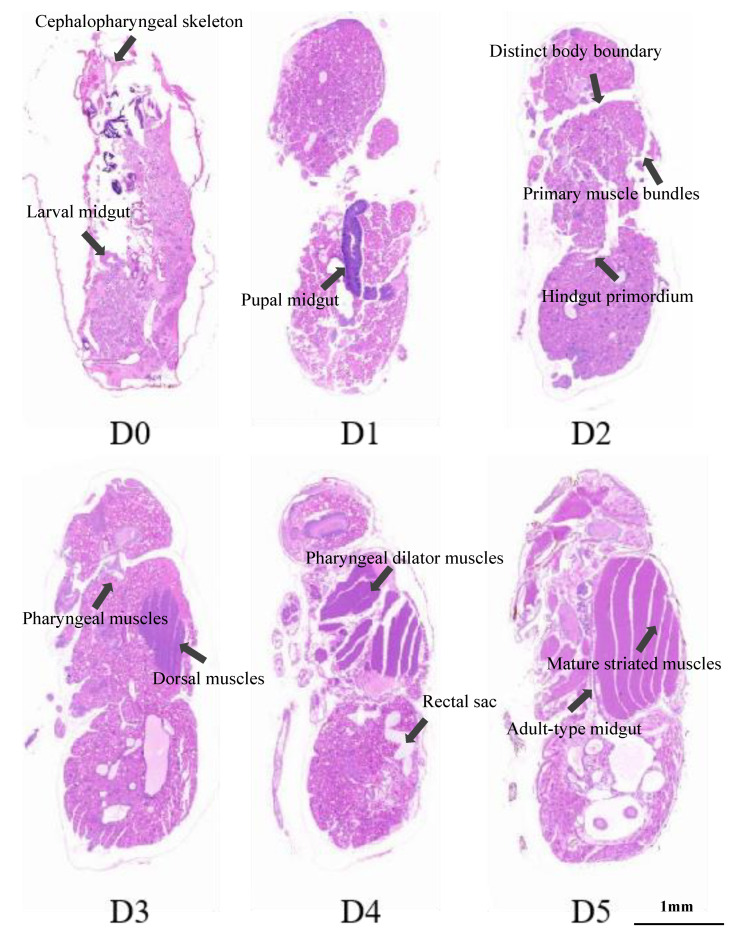
H&E-stained tissue sections of *Phormia regina* pupae at intra-puparial ages D0 to D5 (**D0**–**D5**). Scale bars = 1 mm.

Through HE-stained section observations of *P. regina* pupae at six developmental stages (D0, D1, D2, D3, D4, D5), this study systematically revealed the tissue and organ reconstruction process during the intra-puparial period. Characteristics of each stage are shown in Figure 3. 

D0 Stage: The pupal body exhibited a typical nascent tissue structure, with cells densely and homogenously distributed, without the formation of distinct clear tissue zones. The larval cephalopharyngeal skeleton was still observable at the anterior of the pupa. The coiled, hollow larval midgut remained in the mid-lower part. The epidermis retained subcuticular muscle bundles. Overall, it presented the initial state of tissue remodeling.

D1 Stage: Head, thoracic, and abdominal segments began to differentiate but boundaries were unclear. The midgut structure changed significantly, with the closed, sac-like midgut enlarging to occupy the central position of the body cavity. The muscular system did not yet show specific structures.

D2 Stage: Head, thoracic, and abdominal segments had distinct boundaries. The midgut structure underwent regional contraction and differentiated into the hindgut primordium. The muscular system entered the developmental phase, with locally visible parallel bundles of primary muscle fibers, but striated structures had not yet formed.

D3 Stage: Dorsal muscle groups began to show characteristic striated structures, with muscle fiber bundles arranged orderly. The anterior part of the midgut showed lumen constriction. Pharyngeal muscle groups in the head and thorax began to appear, marking the initial construction of the adult motor system.

D4 Stage: Pharyngeal dilator muscles formed complete bundles. The abdominal rectal sac reached its maximum developmental volume. Various organ systems completed morphological construction. Muscle fiber diameter increased significantly with deep eosinophilic staining, indicating the acquisition of tissue function.

D5 Stage: Muscle fiber bundles reached mature structural state, with clear, densely arranged striations. All system organs completed final differentiation. The midgut completed its transformation into the adult form. The body cavity formed complete functional partitions, morphologically preparing the pupa for eclosion.

### 3.4. Transcriptome Sequencing Results and Data Quality

RNA was extracted from 18 fly pupae. Detection by the 2100 Bioanalyzer showed that the RIN values of all 18 RNA samples were greater than 7.0, meeting the quality requirements for library construction. Transcriptome sequencing of *P. regina* was performed using both PacBio RS II and Illumina platforms. PacBio sequencing generated 47.58 Gb of data. Full-length transcriptome sequencing yielded 537,183 polished Circular Consensus Sequencing (CCS) reads, including 425,349 Full-Length Non-Chimeric (FLNC) sequences. Clustering of FLNC sequences produced 129,155 consensus sequences, with 129,097 being high-quality consensus sequences. Redundancy removal analysis of the high-quality consensus sequences yielded 84,852 transcript sequences. Additionally, Illumina sequencing data also demonstrated high quality.

The results indicated that a total of 38,379 open reading frames (ORFs) were identified in *P. regina*, among which 27,487 were complete ORFs. Simple sequence repeats (SSRs), which serve as important molecular markers in most organisms, were also detected, with 27,168 SSRs identified in this study. Long non-coding RNAs (lncRNAs) were predicted using a combination of four computational methods—CPC, CNCI, CPAT, and Pfam—yielding 50,283 lncRNAs. Additionally, 46,325 transcripts were functionally annotated using the NR, Pfam, KOG/COG/eggNOG, Swiss-Prot, GO, and KEGG databases. Transcriptome sequencing was performed on the Illumina Hiseq4000 platform, and raw sequencing data were filtered to obtain high-quality clean data for subsequent analysis. Statistical analysis of the quality-trimmed sequences showed that the percentage of bases with a quality score of Q30 or higher was 85%, indicating that the sequencing data were of high quality.

### 3.5. Principal Component Analysis (PCA)

**Figure 4:**
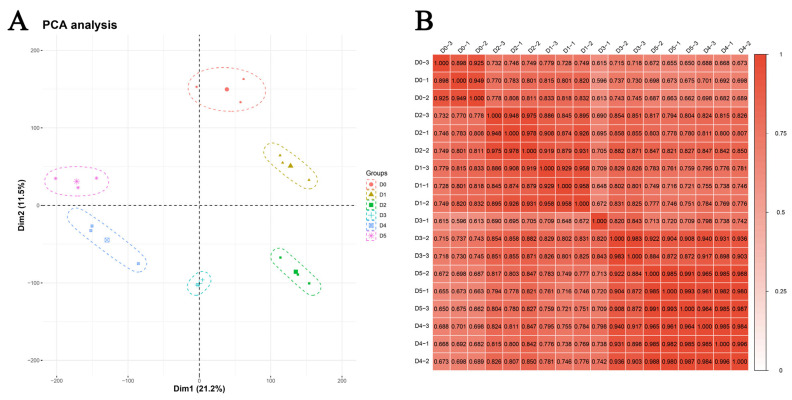
Transcriptomic data analysis across six developmental stages (D0–D5) of *Phormia regina*. (**A**) Principal component analysis (PCA); Dim1 and Dim2 explained 21.2% and 11.5% of total variance, respectively. (**B**) Sample correlation heatmap.

To assess transcriptomic differences among *P. regina* puparia of different ages, PCA was performed. The results showed clear separation of samples from the six developmental stages (Figure 4A).

The Pearson correlation coefficient was used to assess the correlation between biological replicates. An r^2^ value closer to 1 indicates a stronger correlation between two replicate samples. Pearson correlation coefficient analysis across all samples verified the consistency of biological replicates within each developmental stage (Figure 4B).

### 3.6. Differential Gene Expression Analysis

**Figure 5:**
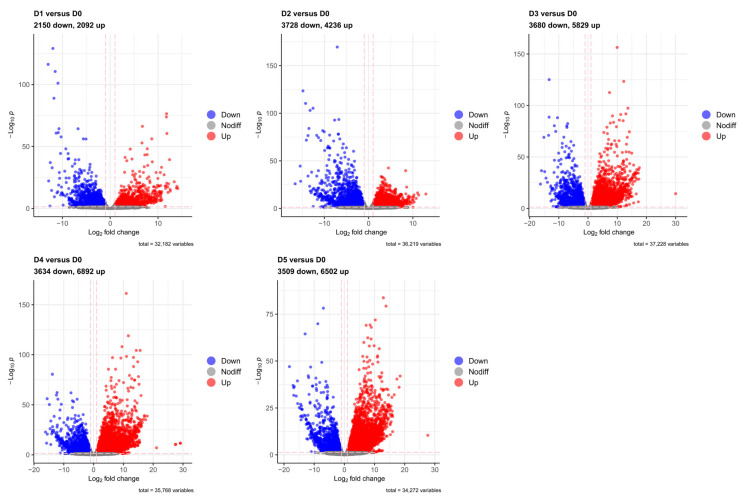
Volcano plots illustrating differentially expressed genes (DEGs) from pairwise comparisons of intra-puparial developmental stages (D1 vs. D0, D2 vs. D0, D3 vs. D0, D4 vs. D0, D5 vs. D0) in *Phormia regina*. Red dots represent significantly upregulated genes, blue dots represent significantly downregulated genes, and gray dots denote non-differentially expressed genes. The number of upregulated and downregulated DEGs for each comparison is labelled at the top of each panel. Vertical dashed lines indicate |log2(fold change)| = 1, and the horizontal dashed line marks—log10(padj) ≈ 1.30 (padj = 0.05). The total number of detected transcripts is labelled at the bottom of each plot, representing the sum of upregulated, downregulated, and non-differentially expressed genes.

Based on 84,852 transcript sequences, this study used the day-0 puparia (D0) as the control group to analyze differential gene expression across samples collected at different postmortem intervals (PMI). Specifically, comparisons were made between D1 vs. D0, D2 vs. D0, D3 vs. D0, D4 vs. D0, and D5 vs. D0. Differential expression was visualized using volcano plots, with significance displayed on the *y*-axis and fold change on the *x*-axis. Significance thresholds were set at a fold change of |log_2_FC| ≥ 1 and an adjusted *p*-value ≤ 0.05 (Figure 5).

The results revealed the following numbers of differentially expressed genes (DEGs): 4242 DEGs (2092 up-regulated, 2150 down-regulated) in D1 vs. D0; 7964 DEGs (4236 up-regulated, 3728 down-regulated) in D2 vs. D0; 9509 DEGs (5829 up-regulated, 3680 down-regulated) in D3 vs. D0; 10,526 DEGs (6892 up-regulated, 3634 down-regulated) in D4 vs. D0; and 10,011 DEGs (6502 up-regulated, 3509 down-regulated) in D5 vs. D0 in *P. regina*.

**Figure 6:**
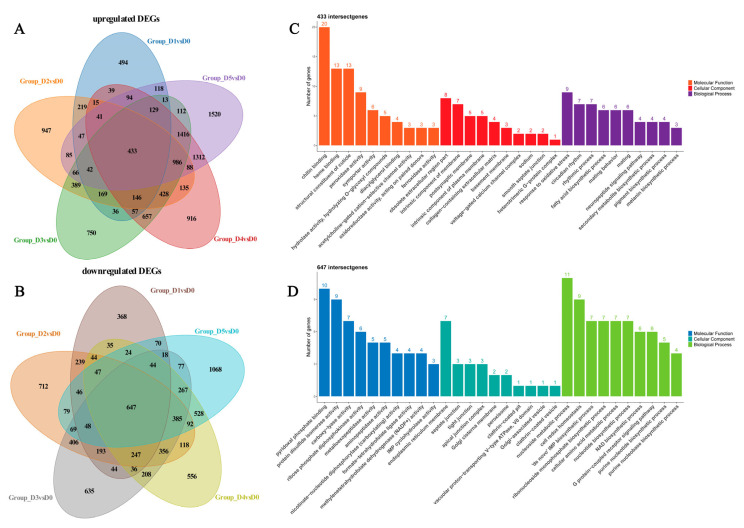
Shared DEGs and GO enrichment of shared genes across D1–D5 vs. D0 comparisons in *Phormia regina*. (**A**) Venn plot of upregulated DEGs; 433 universal upregulated genes were identified; (**B**) Venn plot of downregulated DEGs; 647 universal downregulated genes were identified; (**C**) GO enrichment bar graph of the 433 shared upregulated DEGs (orange: Molecular Function; red: Cellular Component; purple: Biological Process); (**D**) GO enrichment bar graph of the 647 shared downregulated DEGs (blue: Molecular Function; cyan: Cellular Component; green: Biological Process).

Venn diagram (A) in Figure 6 displays the distribution of up-regulated differentially expressed genes (DEGs) across the five comparison groups. A total of 433 genes were commonly up-regulated in all groups. GO enrichment analysis was performed on these 433 shared genes (C). In the Molecular Function category, the results indicate that these genes were significantly enriched for binding and catalytic activity. Within the Cellular Component category, they were predominantly localized to membrane systems and cell junctions. In the Biological Process category, they were primarily involved in metabolic processes, stress response, and the regulation of behavioral rhythms.

Venn diagram (B) in Figure 6 illustrates the distribution of down-regulated differentially expressed genes (DEGs) across the five comparative groups. A total of 647 genes were consistently down-regulated in all groups. GO enrichment analysis of these 647 shared genes (D) revealed significant enrichment patterns. In terms of Molecular Function, genes were primarily associated with various enzymatic activities, such as synthase and lyase activities. Within the Cellular Component category, they were mainly localized to the endomembrane system—including the endoplasmic reticulum and Golgi apparatus—as well as cell junctions. For Biological Processes, genes were predominantly enriched in fundamental biosynthetic and metabolic pathways, such as nucleotide and amino acid anabolism. These findings collectively suggest a marked suppression of essential cellular biosynthesis and metabolic activities.

**Figure 7:**
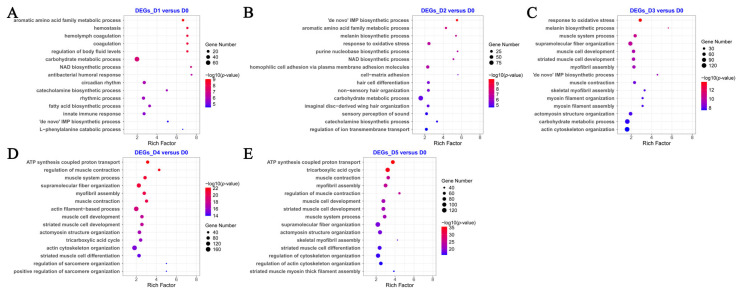
Gene Ontology (GO) enrichment analysis of differentially expressed genes (DEGs) in comparative groups of *Phormia regina*. (**A**) D1 vs. D0, (**B**) D2 vs. D0, (**C**) D3 vs. D0, (**D**) D4 vs. D0, (**E**) D5 vs. D0.

To elucidate the potential biological functions involved, Gene Ontology (GO) enrichment analysis was performed on all differentially expressed genes (DEGs). The results revealed distinct enrichment patterns across the developmental comparisons. In the D1 vs. D0 group, significantly enriched biological processes included aromatic amino acid family metabolic processes, hemolymph coagulation, and blood coagulation. For the D2 vs. D0 group, significantly enriched biological processes involved “de novo” IMP biosynthetic processes, aromatic amino acid family metabolic processes, and melanin biosynthetic processes. In the D3 vs. D0 group, significantly enriched biological processes encompassed the response to oxidative stress, melanin biosynthetic processes, and muscle system processes. Within the D4 vs. D0 group, significantly enriched biological processes comprised ATP synthesis-coupled proton transport, regulation of muscle contraction, and muscle system processes. Finally, in the D5 vs. D0 group, significantly enriched biological processes included ATP synthesis-coupled proton transport, the tricarboxylic acid (TCA) cycle, and muscle contraction (Figure 7).

**Figure 8:**
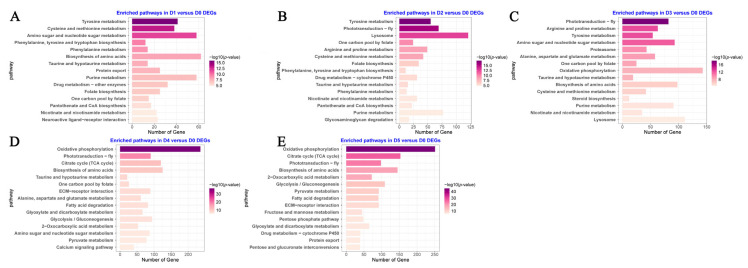
KEGG pathway enrichment analysis of differentially expressed genes (DEGs) in comparative groups of *Phormia regina*. (**A**) D1 vs. D0, (**B**) D2 vs. D0, (**C**) D3 vs. D0, (**D**) D4 vs. D0, (**E**) D5 vs. D0.

KEGG pathway enrichment analysis of the differentially expressed genes (DEGs) revealed distinct patterns across different developmental stages. In the D1 vs. D0 group, DEGs were significantly enriched in amino acid and small molecule metabolic pathways, including tyrosine metabolism, cysteine and methionine metabolism, and amino sugar and nucleotide sugar metabolism. In the D2 vs. D0 group, DEGs were primarily associated with pathways such as tyrosine metabolism, phototransduction, and cytochrome P450-mediated metabolism. For the D3 vs. D0 group, DEGs were enriched in signaling and metabolic pathways including phototransduction, arginine and proline metabolism, and lysosomal pathways. In the D4 vs. D0 group, DEGs showed significant enrichment in energy metabolism and signaling pathways, notably oxidative phosphorylation, phototransduction, and the tricarboxylic acid (TCA) cycle. In the D5 vs. D0 group, DEGs were highly enriched in core mitochondrial energy metabolism pathways such as oxidative phosphorylation, the TCA cycle, and pyruvate metabolism. Notably, the number of enriched genes and statistical significance of these pathways progressively increased from D1 to D5 (Figure 8).

### 3.7. Time Series Analysis

Time-series clustering was performed with the Mfuzz R package (v2.69.0) based on a total of 18,637 unique differentially expressed genes; genes with extremely low expression or negligible temporal fluctuation across sampling points were filtered out according to their expression standard deviation, and Euclidean distance was adopted to quantify the dissimilarity between individual genes.

**Figure 9:**
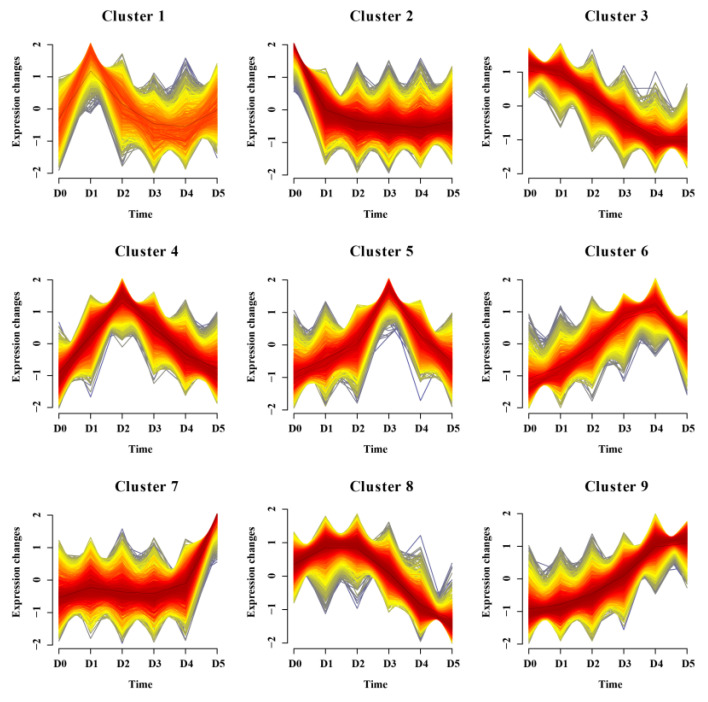
Time-series clustering analysis of intra-puparial developmental transcriptomes in *Phormia regina*. The color gradient from deep red through orange to yellow indicates the density of overlapping gene curves: red regions denote high gene density (most genes share this expression level at the corresponding time point), while yellow regions represent low gene density (fewer genes overlap here). The central band of warm colors reflects the dominant average expression trend of all genes in each cluster.

The optimal cluster number (k = 9) was statistically determined via the Dmin elbow rule: Dmin refers to the minimal inter-centroid distance among all clusters and gradually decreases with the increase in cluster quantity. The obvious elbow inflection point appeared at (k = 9), which realized a proper trade-off between compact intra-cluster gene expression patterns and divergent inter-cluster temporal trends, avoiding under-clustering caused by insufficient grouping or over-fragmentation from excessive cluster division. The fuzzifier parameter was optimized to 1.71186 via functional evaluation. After fuzzy clustering, the acore function was applied to calculate the core membership score of each gene within its assigned cluster, with a minimum acore threshold (min.acore) rationally set to 0.5. Only genes with an acore score ≥ 0.5 were retained as core genes of the corresponding clusters, which excluded genes with ambiguous expression tendencies distributed across multiple clusters and removed noisy hybrid transcripts that interfered with cluster-specific expression patterns. The core function was further used to select representative genes for each cluster relying on membership scores (MEM.SHIP), and the clustering results presented in Figure 9 reveal that these differentially expressed genes were grouped into nine distinct clusters with divergent temporal expression patterns spanning D0 to D5 during intra-puparial development, demonstrating that transcriptional changes underlying pupal metamorphosis are tightly programmed rather than randomly regulated.

**Figure 10:**
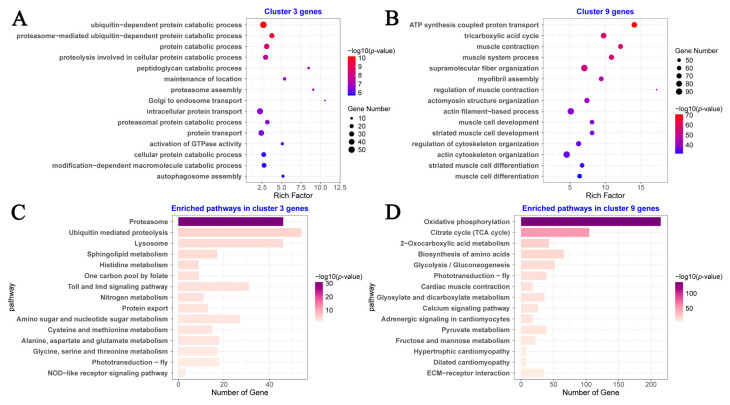
GO and KEGG enrichment analyses for core genes of Cluster 3 (persistently downregulated) and Cluster 9 (persistently upregulated. (**A**,**B**) show bubble plots of the top enriched GO biological process terms for Cluster 3 and Cluster 9. (**C**,**D**) display bar plots of the top enriched KEGG pathways for Cluster 3 and Cluster 9.

Given our core research goal focused on forensic entomological PMI estimation, we selectively carried out GO and KEGG enrichment analyses on Cluster 3 and Cluster 9 only. Genes with continuous upregulation or continuous downregulation display expression levels closely matched to fixed developmental time points, and their stable time-dependent transcriptional signatures are ideal candidate molecular markers to improve the accuracy of PMI inference. Cluster 3 corresponds to persistently downregulated genes over developmental stages, whereas Cluster 9 consists of continuously upregulated genes from D0 to D5. As displayed in Figure 10A,C, core genes of Cluster 3 were prominently enriched in GO terms related to ubiquitin-mediated protein hydrolysis, cellular protein catabolic process and endosome transport, alongside significantly enriched KEGG pathways including proteasome, lysosome and ubiquitin-regulated proteolysis, indicating critical roles of protein degradation and tissue remodeling at the early puparial developmental stage. Conversely, core genes from Cluster 9 (Figure 10B,D) were mainly enriched in GO entries involved in mitochondrial energy metabolism, tricarboxylic acid cycle and muscle cell differentiation, matching enriched KEGG pathways of oxidative phosphorylation, citrate cycle and muscle contraction, which reflects enhanced energy supply and adult muscle formation during late intra-puparial maturation.

### 3.8. Validation of Gene Expression Levels

**Figure 11:**
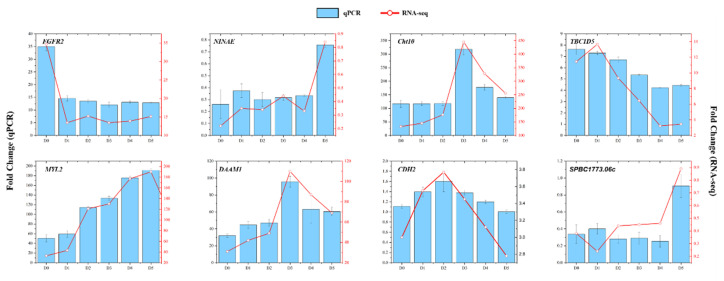
Validation of expression levels of eight selected genes by quantitative PCR (qPCR) during intra-puparial development of *Phormia regina*. Blue bars: qRT-PCR expression fold change (left Y-axis); red line: RNA-seq expression fold change (right Y-axis). The parallel expression trajectories confirm the consistency of RNA-seq and qRT-PCR results.

To validate the results obtained from RNA-seq analysis, this study selected eight genes associated with visual and muscular development and designed specific primers for quantitative PCR (qPCR). The expression levels of these genes were compared across developmental stages from D0 to D5. The qPCR results demonstrated that the expression patterns of these eight genes were consistent with those identified by RNA-seq, confirming the reliability of the sequencing data (Figure 11).

## 4. Discussion

Within the immature stage of *P. regina*, the intra-puparial period is the longest in duration [13]. Based on the measured average body weight data, body mass tended to drop rapidly from D0 to D1 before entering a slower decreasing phase across subsequent developmental days. The average total weight declined from 3.2650 g at D0 to 2.9337 g at D1, with an absolute weight loss of 0.3313 g, which accounted for approximately 70% of the total 0.4742 g weight loss throughout D0–D5. Extensive larval tissue histolysis and organ remodeling proceed inside the puparium in early intra-puparial development, accompanied by high energy expenditure and metabolic waste accumulation [31]. From D1 to D5, average body weight decreased moderately from 2.9337 g to 2.7908 g (total loss = 0.1429 g), showing a slower daily weight reduction relative to the D0–D1 interval. This trend may be related to the completion of primary tissue reconstruction and progressive maturation of muscle and endocrine systems during mid and late metamorphosis [32]. Cepeda-Palacios and Scholl [33] reported a similar early-dominated weight-loss pattern in *Oestrus ovis*, with 23% body weight lost within the first week of pupal development versus 4% and 7% weekly loss in the following three weeks.

Ma et al. [19] suggested that internal changes during intra-puparial morphogenesis may serve as better age indicators. We observed that at the onset of morphogenesis, internal intra-puparial structures were fragile and adhered to the puparium. From D1 to D2, the basic adult structures (head, thorax, abdomen, legs, wings, antennae, mouthparts) formed rapidly and became more defined. By D3, external structures (e.g., wing folding, bristles, setae, abdominal segmentation) were fully developed, and morphology stabilized. From D4 to D5, extensive pigment synthesis and deposition occurred, leading to rapid darkening of body color in preparation for eclosion. Richards et al. [17] identified wing emergence and leg development as common thoracic characteristics used in intra-puparial developmental staging. Cepeda-Palacios and Scholl [33] considered segmentation, color change, and spiracle development as the most notable abdominal features. Karabey and Sert [20], through observations of intra-puparial developmental stages, noted that, in addition to structural changes such as leg and wing development, stigmatic openings on the abdomen and mouthparts, and thinning/shedding of the pupal cuticle, all stages were associated with coloration of body parts. In this study, we observed that compound eyes were the first to pigment (D4), followed by the bristles and setae on the head and thorax as well as the edges of the legs (D4). Finally, by D5, structures such as antennae, labrum, wings, and legs darkened to a dark brown or deep gray. This sequence aligns with pigmentation patterns recorded in calliphorid and muscid species by Flissak and Moura [21], Salazar-Souza et al. [22], do Couto and Queiroz [23], Duarte and Queiroz [24], and Sert and Ergil [17]. These morphological characteristics constitute promising phenotypic markers for intra-puparial developmental staging and PMI estimation in forensic investigations. Nevertheless, pigmentation progression and developmental rates are susceptible to ambient temperature as well as intraspecific geographic population variation. Considering the fluctuating thermal conditions encountered at actual forensic crime scenes, additional validation experiments under variable temperature regimes and with geographically distinct populations are necessary prior to the routine application of these morphological markers in forensic casework.

To elucidate the molecular regulatory mechanisms underlying different developmental phases of *P. regina* intra-puparial development, this study employed, for the first time, a combined second- and third-generation sequencing strategy. Full-length transcriptome information was obtained from *P. regina* puparia reared under constant 25 °C conditions. By screening differentially expressed genes (DEGs) across stages and performing GO and KEGG enrichment analyses, we aimed to reveal the biological functions and pathways associated with these DEGs, providing a theoretical basis for understanding stage-specific molecular mechanisms and behavioral traits during intra-puparial development.

Stage-specific DEGs play crucial roles in regulating growth and development [34], and their expression patterns exhibit significant dynamic changes throughout development [35]. In this study, comparative analyses identified 4242 DEGs for D1 vs. D0, 4686 for D2 vs. D1, 4881 for D3 vs. D2, 3827 for D4 vs. D3, and 2918 for D5 vs. D4. The number of DEGs increased continuously during intra-puparial development before stabilizing in later stages, which may be closely related to the regulatory mechanisms governing this phase.

Building on this foundation, the time-series clustering analysis further elucidated the stage-specific regulatory patterns of gene expression. In Cluster 9 (Figure 9), we observed a steady increase in the expression of skeletal muscle protein genes throughout *P. regina* intra-puparial development. This finding is highly consistent with the remodeling and development of muscular tissues during insect metamorphosis. The intra-puparial period is a critical phase for the transition from larva to adult, involving complex tissue reorganization. The sustained upregulation of skeletal muscle protein genes likely reflects the progressive formation of the adult muscular system, particularly the thoracic muscles associated with flight [36]. In dipterans, the indirect flight muscles (IFMs) constitute the largest and most powerful muscle group, and their development spans the entire intra-puparial stage [37]. Reedy and Beall [31] investigated muscle development in *Sarcophaga bullata* puparia and reported that IFM development begins early in pupariation, but major myofiber growth and maturation occur during the latter half, aligning with our observed trend of increasing skeletal muscle gene expression. Conversely, autophagy-related genes in Cluster 3 exhibited a steady decline throughout development. Autophagy is a key mechanism for tissue remodeling during insect metamorphosis, particularly responsible for degrading larval tissues in the early intra-puparial period [38]. Neufeld [39], in Drosophila studies, found high expression of autophagy-related genes (e.g., Atg1, Atg8a) early in pupariation, followed by a gradual decrease, consistent with our observations in *P. regina.* The continued decline in autophagy gene expression likely signifies a transition from tissue degradation to tissue reconstruction. In early pupariation, extensive larval tissue is broken down to provide energy and raw materials for adult tissue development [40]. As development proceeds and new adult tissues form, autophagic activity diminishes accordingly [41]. These stable increasing or decreasing expression trends provide valuable molecular markers for distinguishing between early and late intra-puparial periods.

In Cluster 7, we found that the opsin Rh1 visual protein gene maintained low-level expression from D0 to D4 but increased significantly from D4 to D5. This expression pattern aligns closely with the observed late-stage eye pigmentation. Opsin Rh1 is a key protein in phototransduction, and its increased expression likely signifies eye maturation [42]. We observed a significant increase in opsin Rh1 expression during the late intra-puparial period of *P. regina*, ensuring the normal function of the visual system upon adult emergence. This notable change in expression provides an effective molecular marker for distinguishing between mid- and late-stage puparia. Morphogenesis-related activator genes in Cluster 5 peaked in expression at D3 while remaining low from D0–D2 and D4–D5. This pattern corresponds to the critical period of wing morphogenesis, providing a molecular marker for identifying mid-stage puparia. Insect wing development is a complex process involving spatiotemporally specific expression of multiple genes [43]. Celis [44], investigating wing development in Drosophila, found that several key transcription factors were highly expressed within specific developmental time windows, with their expression peaks corresponding to critical phases of wing morphogenesis. Compared with morphological studies on *P. regina* puparia, the D3 expression peak we observed reflects the same developmental process.

RPL23, a ribosomal protein, has been validated as a stable reference gene in multiple studies [45]. qPCR validation using RPL23 as the reference gene showed high consistency with our transcriptome data, further confirming the reliability of our findings. The differentially expressed genes and expression patterns identified in this study offer multiple potential molecular markers for developing more precise methods of intra-puparial age estimation. For example, combining expression levels of skeletal muscle and autophagy genes could enable fine subdivision of the entire intra-puparial period. Meanwhile, expression changes in opsin Rh1 and morphogenesis-related activators could be used to identify specific developmental stages. These findings significantly enhance the accuracy of PMI estimation based on *P. regina* puparia [46]. However, it must be emphasized that these findings should be interpreted as exploratory correlative results rather than validated forensic tools. Therefore, these genes should currently be regarded as candidate developmental markers that require validation across different temperatures, geographic populations, fluctuating environmental conditions, and independent datasets before their application in forensic practice can be considered. Future studies should integrate proteomic and metabolomic data and conduct similar research under varying temperatures to obtain a more comprehensive developmental atlas of the intra-puparial period.

In this study, we integrated morphological observation, histological sectioning, intra-puparial weight measurement, and full-length transcriptome sequencing to characterize the intra-puparial development of *P*. *regina* at multiple biological levels. Morphological and histological observations visualized tissue remodeling and organogenesis, while intra-puparial weight dynamics reflected metabolic and energy expenditure characteristics, with transcriptomic analysis further revealing the core molecular regulatory networks driving developmental changes. By associating the expression of genes related to skeletal muscle formation, autophagy, phototransduction and morphogenesis with typical intra-puparial morphological traits such as eye pigmentation, wing folding and muscle development, we established a comprehensive molecular-morphological framework for high-resolution intra-puparial age estimation. This multi-indicator strategy substantially improves the accuracy and robustness of PMI estimation. Morphological characteristics allow rapid on-site preliminary staging, whereas stage-specific molecular markers provide high-temporal-resolution quantitative references. The combination of multiple independent indicators effectively reduces the inherent bias of single-method evaluation. Compared with traditional morphological methods that only achieve daily-scale resolution, the integration of qPCR-based molecular detection further refines intra-puparial age discrimination to an hourly or higher precision.

## 5. Conclusions

This study systematically investigated the dynamic developmental processes of *P. regina* puparia at 25 °C through an integrated morphological and full-length transcriptomic analysis. Morphologically, a detailed internal and histological developmental atlas of *P. regina* puparia was successfully constructed. Meanwhile, an overall decreasing trend in body weight during intra-puparial period was observed, with the most significant decline occurring during the initial phase of pupariation. At the transcriptomic level, for the first time, a combined second- and third-generation sequencing approach was applied to analyze six developmental stages (0–5 days) of *P. regina* puparia. This approach not only identified differentially expressed genes (DEGs) between successive developmental stages but also elucidated the expression patterns of key genes, such as those encoding skeletal muscle proteins. The reliability of the sequencing data was further confirmed by quantitative real-time PCR (qPCR) results. In summary, this study provides a clear elucidation of the morphological characteristics and molecular regulatory mechanisms underlying intra-puparial development in *P. regina.* These findings offer a more precise scientific foundation for estimating the postmortem interval (PMI) and analyzing insect evidence in the field of forensic science.

Nevertheless, this study has several limitations that should be considered when interpreting the results. First, all experiments were performed at a constant 25 °C temperature, yet ambient temperatures at actual forensic scenes fluctuate continuously. Thus, our morphological data and candidate reference gene expression profiles serve solely as baseline references, with further testing under variable thermal regimes needed to evaluate both morphological outcomes and marker gene expression stability. Second, this study used only a single laboratory population, and the cross-population robustness of the candidate markers needs to be validated through comparative studies across multiple geographic populations. Third, this study screened stage-specific differentially expressed genes of *P. regina* by transcriptome sequencing, but their in vivo and in vitro regulatory mechanisms have not been verified experimentally. In subsequent research, we will perform relevant functional tests on representative key genes to further clarify their regulatory functions during intra-puparial development. Fourth, the proposed morphological and molecular markers should currently be regarded as candidate markers, as their predictive accuracy has not been tested in independent datasets nor their practical utility validated in real-world scenarios.

This study establishes a distinct qualitative staging system based on intra-puparial morphological observation. Further research will adopt quantitative imaging methods such as colorimetry and morphometry to achieve more objective and intelligent age estimation in forensic practice. Studies will also be carried out to verify markers under varying temperatures and conduct comparative analysis among different populations. Combined with multi-omics data and machine learning algorithms, high-precision age prediction models will be constructed, advancing forensic entomology from descriptive research to predictive research.

## Figures and Tables

**Table 1:** Primers used for qRT-PCR.

Gene ID	Gene Description	Primer Name	Primer (5′-3′)	Length/bp
FY-mixed_transcript_36495	60S ribosomal protein L23	RPL23	F: GTGATAGTGAATAACAAGGGTG R: AGAACTGGCATTTGAGGC	105
FY-mixed_transcript_113211	myosin regulatory light chain 2	MYL2	F: AGCACCTGCCTCCACTAC R: TTGCCAATGATACCATCC	171
FY-mixed_transcript_126472	chitinase 10	Cht10	F: AGTATGGGATTCCGAACAAC R: ACCACGAGATTCATAGGGAC	263
FY-mixed_transcript_59974	neural-cadherin	CDH2	F: TCGTTTACATTTCCGCTAC R: CCTCCTGTTTATCAACCC	220
FY-mixed_transcript_122608	opsin Rh1	NINAE	F: TGGCAAAGTTGATGATGG R: GATACACGAGGTGCTGTT	117
FY-mixed_transcript_14367	zinc-type alcohol dehydrogenase-like protein C1773.06c	SPBC1773.06c	F: GATGAGGGCTTCCGTTTA R: CGATGAAGACTGCGTGAA	161
FY-mixed_transcript_127574	TBC1 domain family member 5	TBC1D5	F: TGCCCACATCTGACACTG R: TATCGCATCCCACAGCAC	233
FY-mixed_transcript_9831	Fibroblast growth factor receptor-like protein 2	FGFR2	F: GTCGCTTAGGTGTTGATA R: CCTTGTATGCGGTTATTT	205
FY-mixed_transcript_73273	disheveled-associated activator of morphogenesis 1	DAAM1	F: GGGGAGGTAAACTCAACA R: CAATCTCCATAACGCAAA	246

## Data Availability

Data will be made available on request.
